# Supplementary material for: Bioreactance assessment of cardiac output lacks reliability for the follow-up of patients with pulmonary hypertension
Source: PLoS One. 2024 May 20;19(5):e0298727. doi: 10.1371/journal.pone.0298727 (PMC11104646; doi:10.1371/journal.pone.0298727)
Supplement: Attachment — Submitted filename: Response to reviewer.docx [file pone.0298727.s001.docx]

Dear Pr Karhausen,

Please find appended the revised version of the manuscript entitled “Bioreactance assessment of cardiac output lacks reliability for the follow-up of patients with pulmonary hypertension” (one version with modifications made apparent, and a second clean version), and below a point-by-point reply to comments made.

We hope that this version is acceptable for publication in Plos One, but remain at your disposal if any further modifications are required.

Thank you for your consideration,

Yours sincerely

Ségolène Turquier, on behalf of the co-authors.

**Editor comments.**

1. Line 38 “To date, only one study was conducted in a cohort of 50 patients, PH.” -> with PH?
2. Line 94: “Precapillary PH belonged for 75% to group”-> precapillary PH made up 75% of the group?
3. Please describe what clinical classification groups 1,4,5 stands for.
4. Please clarify abstract, it states that 110 datasets were analyzed. While this is correct, a better representation of the work would be to say that 60 PH patients were included with full datasets available for 50patients on follow up.

**Authors:**

1. Revised accordingly, line 61 “with PH”

2 and 3. As requested we have changed the sentence, lines 115-122: “In accordance with the last clinical classification of PH, 75% (n=45/60) of cases were attributed to group 1 (pulmonary arterial hypertension) (1). Among these, the distribution was as follows: 31.7% idiopathic; 5.0% heritable; 6.7% associated with drug; 15% associated with connective tissue disease; 3.3% associated with operated congenital heart disease; 1.7% associated with human immunodeficiency virus infection; 8.3% associated with portal hypertension; 3.3% pulmonary veno-occlusive disease. A total of 20% (n=12/60) of patients were in group 4 (PH associated with pulmonary artery obstructions) and 5% (n= 3/60) in group 5 (PH with unclear and/or multifactorial mechanisms).”

4. Following this comment, we have changed the sentence, lines 36, 37: “A total of 60 PH patients were included. All datasets were available at the baseline visit (V0) and 50 of them were usable during the follow-up visit (V1).”

**Reviewer comments.**

1. My only suggestion for revision is to add to the discussion regarding the gold standard of cardiac output assessment. … The authors say they avoided the pitfalls of TD measurement but these are inherent to the method and I am not sure how they can avoid they completely.
2. I think the ultimate conclusion - that the device is not adequate for follow up of PH patients - still holds based on their data, but they need to acknowledge that the gold standard they are using is also prone to error

**Authors:**

1 And 2. We concur with the reviewer's assessment that no CO measurement technique, whether invasive or non-invasive, is perfectly reliable. Following this comment we have added :

lines 189-191: “Due to the complexity associated with the time and equipment required for direct Fick CO measurement, most PH centers prefer to utilize the thermodilution technique.”

Line 225, 226: “thermodilution, according to the guidelines, is the gold standard used in the majority of PH centers, including our own”

Line 228-230: “Unfortunately, to date, no CO measurement technique is perfectly accurate. Aware of these pitfalls, we have made effort to avoid potential problems, to the best of our abilities.”

In lines 226-228, we discuss the main sources of error in thermodilution, referencing the article authored by AR Opotowsky *et al.* as a source.
